# Intramolecular Hydrogen Bonds Assisted Construction of Planar Tricyclic Structures for Insensitive and Highly Thermostable Energetic Materials

**DOI:** 10.3390/ijms25073910

**Published:** 2024-03-31

**Authors:** Yubing Liu, Jie Li, Jinxiong Cai, Xun Zhang, Lu Hu, Siping Pang, Chunlin He

**Affiliations:** 1 School of Materials Science & Engineering, Beijing Institute of Technology, Beijing 100081, China; liuyb@bit.edu.cn (Y.L.); lijeovg@163.com (J.L.); 7520230186@bit.edu.cn (J.C.); zhangxun@bit.edu.cn (X.Z.); lhu@bit.edu.cn (L.H.); 2 Experimental Center of Advanced Materials, School of Materials Science & Engineering, Beijing Institute of Technology, Beijing 100081, China; 3 Chongqing Innovation Center, Beijing Institute of Technology, Chongqing 401120, China

**Keywords:** energetic compounds, tricyclic frameworks, planar structure, intramolecular hydrogen, stability

## Abstract

Safety is fundamental for the practical development and application of energetic materials. Three tricyclic energetic compounds, namely, 1,3-di(1H-tetrazol-5-yl)-1H-1,2,4-triazol-5-amine (**ATDT**), 5′-nitro-3-(1H-tetrazol-5-yl)-2′H-[1,3′-bi(1,2,4-triazol)]-5-amine (**ATNT**), and 1-(3,4-dinitro-1H-pyrazol-5-yl)-3-(1H-tetrazol-5-yl)-1H-1,2,4-triazol-5-amine (**ATDNP**), were effectively synthesized through a simple two-step synthetic route. The introduction of intramolecular hydrogen bonds resulted in excellent molecular planarity for the three new compounds. Additionally, they exhibit regular crystal packing, leading to numerous intermolecular hydrogen bonds and π–π interactions. Benefiting from planar tricyclic structural features, **ATDT**, **ATNT**, and **ATDNP** are insensitive (*IS* > 60 J, *FS* = 360 N) when exposed to external stimuli. Furthermore, **ATNT** (*T*_d_ = 361.1 °C) and **ATDNP** (*T*_d_ = 317.0 °C) exhibit high decomposition temperatures and satisfying detonation performance. The intermolecular hydrogen bonding that produced this planar tricyclic molecular structure serves as a model for the creation of innovative multiple heterocycle energetic materials with excellent stability.

## 1. Introduction

Energetic materials (EMs), a unique class of functional materials, have contributed significantly to the advancement of human civilization and have played a significant role in advancing human civilization and progress, particularly over the last century [1,2,3,4]. Currently, the application of EMs extends beyond military purposes and is extensively studied in various fields such as aerospace, mining, construction, demolition, and safety equipment [5,6,7]. Particularly, in some specialized fields, greater safety requirements have been proposed, such as an impact sensitivity higher than 40 J and a thermal decomposition temperature exceeding 300 °C [8,9,10]. As a means of meeting these goals, multiple N-heterocycle frameworks have garnered significant attention in recent decades. These frameworks possess abundant intrinsically energetic C−N and N−N bonds, as well as the presence of electron-delocalized planar conjugated structures and pollution-free decomposition products. Furthermore, the diversity of building blocks in multiple heterocycle frameworks is advantageous for integrating the strengths of various skeletons, thereby enhancing overall comprehensive performance [11,12,13,14]. In the pursuit of high-energy and high-stability compounds, various multiple heterocycle energetic compounds have been constructed based on diverse building blocks. For example, Cheng et al. [15,16] successfully utilized the condensation and cyclization reactions between acetohydrazide and ethyl acetimidate to construct 1,2,4-triazole, bridging two pyrazole rings or connecting furazan and pyrazole. The resulting tricyclic energetic compounds often exhibit excellent thermal stability, with initial decomposition temperatures exceeding 300 °C. Additionally, they demonstrate impressive energy performance, with detonation velocities that exceed 8800 m s^−1^. In 2023, Tang et al. [17] employed a silver-catalyzed cyclization reaction to construct 1,2,4-triazole based on a different skeleton, resulting in production of variety of tricyclic energetic compounds. Compounds **DTTN** and **BTTN** have decomposition temperatures of 286 °C and 316 °C, respectively. However, due to the unmodified sites and low density, compounds **DTTN** (*vD*: 7793 m s^−1^) and **BTTN** (*vD*: 6997 m s^−1^) exhibit unsatisfactory detonation performance.

Generally, due to the features of single-bond connections and steric hindrance effects, as the number of rings increases, the multicyclic framework is more likely to display reduced planarity, resulting in a decrease in both the density and stability [18,19,20]. Crystallographic analysis of tricyclic energetic compounds (such as **BDT-2**, **DPTO**, **DNNT**, and **BNNT**) revealed a significant angle between the 1,2,4-triazole and the other two rings in their molecules. Poor molecular planarity leads to impact sensitivity distributed between 22 and 40 J, and the densities of compounds **DNNT** and **BNNT** are also relatively low, at 1.716 g cm^−3^ and 1.562 g cm^−3^, respectively. Furthermore, the synthesis of these compounds usually undergoes two stages: initiating the formation of a new ring through cyclization reactions involving specific functional groups followed by energetic modifications through multistep C-functionalization or N-functionalization (such as the introduction of nitro groups) under strong oxidation conditions [21,22,23]. The complex synthetic routes tend to produce by-products, reducing overall yields. Moreover, challenges may arise in introducing functional groups or experiencing molecular ring-opening hydrolysis due to the low reactivity of certain sites [24,25,26]. Therefore, improving molecular planarity and creating tricyclic energetic compounds through facile and efficient molecular synthesis strategies remain significant challenges that require further exploration.

**Figure 1:**
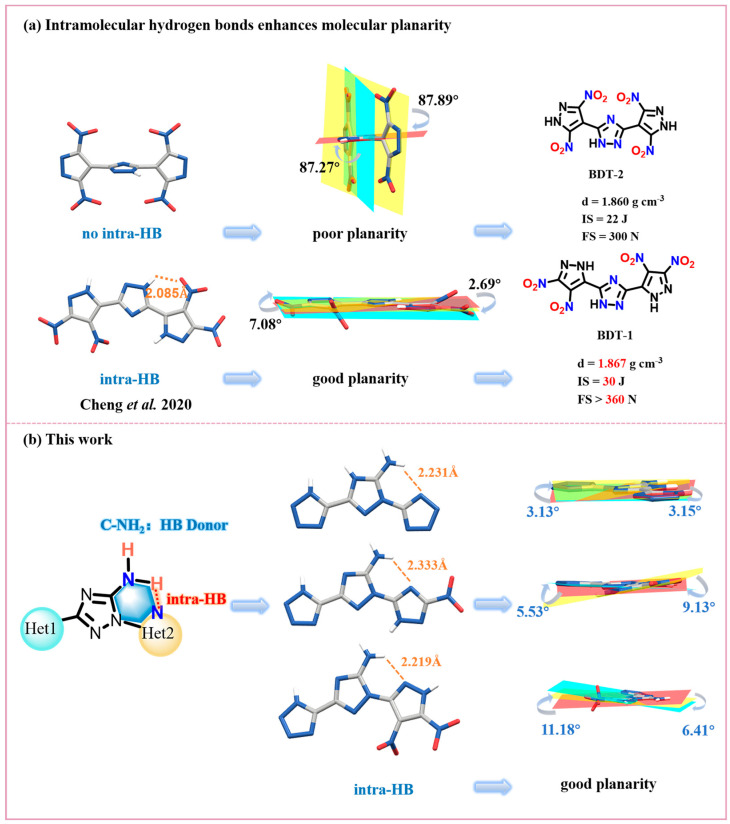
(**a**) Intramolecular hydrogen bonds enhance molecular planarity [15]; (**b**) By designing intramolecular hydrogen bonds, novel planar tricyclics energetic compounds are constructed.

One of the most planar compounds is 1,3,5-triamino-2,4,6-trinitrobenzene (**TATB**), which has been used as a standard model for creating coplanar and insensitive EMs [27,28]. Intramolecular hydrogen bonds (intra-HBs), formed between electron-rich atoms and protons, often along with π-electron delocalization, can enhance molecular planarity and contribute to larger π–π stacking interactions. Hence, the formation of intra-HBs in tricyclic energetic molecules is crucial for enhancing their planarity. As illustrated in Figure 1a, for example, in the case of BDT-2, the molecule lacks intra-HBs, leading to angles between the two pyrazole rings and the 1,2,4-triazole of 87.27° degrees and 87.89°, respectively. Conversely, in the molecule of **BDT-1**, there is a strong intra-HB between C-NO_2_ and NH that results in good molecular planarity, higher density, and lower mechanical sensitivity. However, examples like **BDT-1** are rare, as the functional groups (-NO_2_, -NH_2_ etc.) are commonly distributed on the end rings of tricyclic energetic molecules, and the central ring often lacks hydrogen bond donors or acceptors, making it challenging to form intra-HBs. Additionally, due to steric hindrance effects, nitro groups tend to deviate from the skeleton plane, which presents a challenge for the formation of hydrogen bonds with NH. Based on the above analysis, we designed the tricyclic energetic compounds with amino-1,2,4-triazole as the central component. The presence of C-NH_2_ in the central ring ensures the existence of intra-HBs within the molecule. As envisioned, in this work, successful synthesis and characterization of three tricyclic energetic compounds with improved molecular planarity, high thermal stability, and low sensitivity were achieved (Figure 1b).

## 2. Results and Discussion

### 2.1. Synthesis

**Scheme 1:**
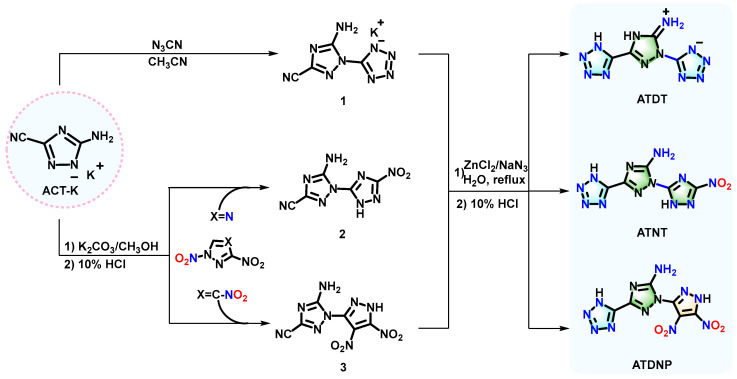
Synthetic route of compounds **ATDT**, **ATNT**, and **ATDNP**.

The starting material, potassium 5-amino-1H-1,2,4-triazole-3-carbonitrile (**ACT-K**), was synthesized in accordance with the available literature [29], as shown in Scheme 1. **ACT-K** was reacted with cyanogen azide to produce intermediate potassium 5-amino-1-(1H-tetrazol-5-yl)-1H-1,2,4-triazole-3-carbonitrile (**1**) (CCDC† 2233193). Subsequently, a [3 + 2] cycloaddition between cyano and azide occurred, obtaining the first target intra-HB-type tricyclic energetic molecule, 1,3-di(1H-tetrazol-5-yl)-1H-1,2,4-triazol-5-amine (**ATDT**). The compound **ATDT** is a CHN-type molecule. To increase the oxygen content of the compounds, we aimed to substitute the tetrazole with nitro-1,2,4-triazole and dinitro-pyrazole. Thus, the intermediate compounds 5-amino-5′-nitro-2′H-[1,3′-bi(1,2,4-triazole)]-3-carbonitrile (**2**) (CCDC† 2244941) and 5-amino-1-(3,4-dinitro-1H-pyrazol-5-yl)-1H-1,2,4-triazole-3-carbonitrile (**3**) (CCDC† 2233192) were obtained by reacting 1,3-dinitro-1,2,4-triaozle or 1,3,4-trinitropyrazole with **ACT-K** in the presence of K_2_CO_3_ in methanol. Subsequently, intermediates **2** and **3** were treated with sodium azide, successfully yielding two additional tricyclic energetic compounds with intra-HBs: 5′-nitro-3-(1H-tetrazol-5-yl)-2′H-[1,3′-bi(1,2,4-triazole)]-5-amine (**ATNT**) and 1-(3,4-dinitro-1H-pyrazol-5-yl)-3-(1H-tetrazol-5-yl)-1H-1,2,4-triazol-5-amine (**ATDNP**). In fact, the more diverse building blocks a molecule contains, the greater the challenge of its synthesis. Notably, **ATDNP** incorporates three different building blocks, marking the first instance of the combination of pyrazole, 1,2,4-triazole, and tetrazole within a tricyclic ring. Elemental analysis, differential scanning calorimetry (DSC), infrared spectroscopy, and multinuclear NMR spectroscopy were used to properly describe all new compounds. The structures of **ATDT**, **ATNT**, and **ATDNP** were confirmed by single-crystal X-ray diffraction.

### 2.2. Single Crystal X-ray Structure Analysis

**Figure 2:**
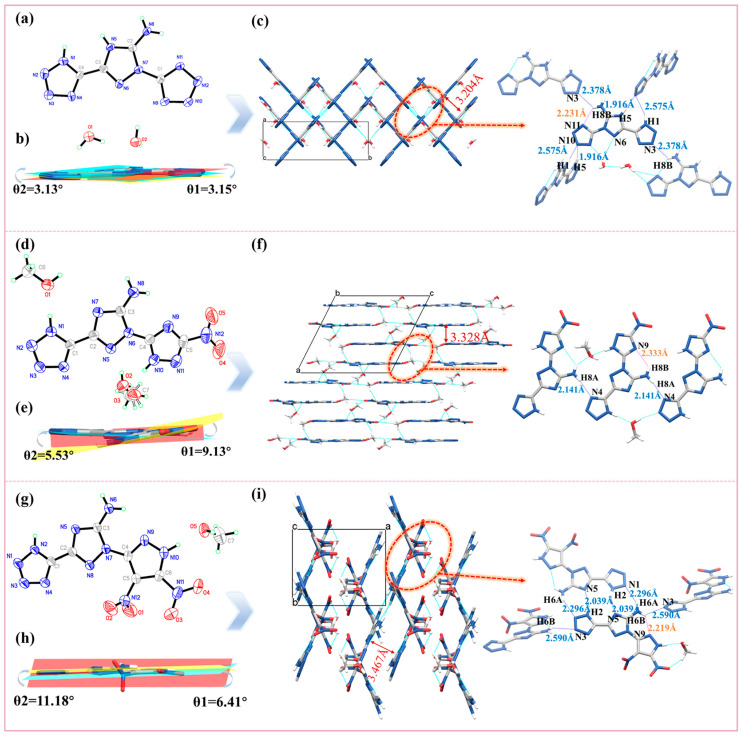
(**a**) Crystal structures of compounds **ATDT**·2H_2_O, (**d**) **ATNT**·2CH_3_OH, and (**g**) **ATDNP**·CH_3_OH; side view of molecules (**b**) **ATDT**, (**e**) **ATNT**, and (**h**) **ATDNP**; the crystal packing diagrams and H-bonds (dashed line) of (**c**) **ATDT**·2H_2_O, (**f**) **ATNT**·2CH_3_OH, and (**i**) **ATDNP**·CH_3_OH.

Single-crystals of **ATDT**, **ATNT**, and **ATDNP** (Figure 2a,d,g) and intermediates **1**–**3** (Figure S1) were determined by single-crystal X-ray diffraction. The compound **ATDT**·2H_2_O (CCDC† 2201504) crystallizes in the monoclinic space group *Cc*, with four independent molecules in each lattice cell (Z = 4), and the crystal density is 1.662 g cm^−3^ at 296 K. With angles of 3.13° and 3.15°, respectively, between the two tetrazole rings and the 5-mine-1,2,4-triazole ring, the two tetrazole rings exhibit a nearly coplanar structure with the 1,2,4-triazole ring. (Figure 2b). Furthermore, the intra-HB between an amino’s hydrogen and a nitrogen in the adjacent rings can be observed with the length of the hydrogen bond (HB) N(8)-H(8B)⋯N(11) measuring 2.231 Å. With a layer spacing of 3.204 Å, the packing diagram of ATDT·2H_2_O displays a cross stacking that is substantially shorter than the geometrical parameters of aromatic π–π interactions (3.65–4.00 Å). A benefit of this packing type is that it is not easily affected by external mechanical stimulus [30]. The bond length of C2-NH_2_ in the 1,2,4-triazole ring is 1.351(5) Å, belonging to the double bond, indicating the formation of a zwitterionic compound. Crystals of **ATNT**·2CH_3_OH (CCDC† 2233195) and **ATDNP**·CH_3_OH (CCDC† 2233194) were obtained by slow evaporation of methanol at room temperature. They crystallize in monoclinic systems with *P21*/*n* and *P21*/*c* space groups, respectively. The calculated densities of the crystals of **ATNT**·2CH_3_OH and **ATDNP**·CH_3_OH are 1.516 and 1.671 g cm^−3^, respectively, at 298 K (Z = 4). The bond length of C4-N6 in **ATNT**·2CH_3_OH is 1.365(4) Å, shorter than C1-N7 (1.397(5) Å) in **ATDT**·2H_2_O and C4-N7 (1.397(3) Å) in **ATDNP**·CH_3_OH. Additionally, the bond length of C5-NO_2_ in crystal **ATNT**·2CH_3_OH is 1.443(3) Å, which is close to C5-NO_2_ (1.451(4) Å) and C6-NO_2_ (1.435(4) Å) in the crystal **ATDNP**·CH_3_OH. From Figure 2e, the angles between the tetrazole ring, N-site-1,2,4-triazole ring and 5-mine-1,2,4-triazole ring are 5.53° and 9.13°, respectively. Figure 2f illustrates the crystal packing of **ATNT**·2CH_3_OH, showing a face-to-face stacking arrangement with a layer spacing of 3.328 Å, indicating strong π–π interactions between molecules. The length of the intra-HB, N(8)-H(8A)⋯N(4) measures 2.141 Å. Figure 2h indicates that the angles between the tetrazole ring, N-pyrazole ring and 5-amine-1,2,4-triazole are 11.18° and 6.41°, respectively. The length of the intra-HB, C3-H(6B)⋯N9, is 2.219 Å. The crystal packing of **ATDNP**·CH_3_OH is a cross-stacking with a layer spacing of 3.467 Å (Figure 2i), falling within the scope of typical geometrical parameters of aromatic π–π interactions. Based on the crystal analysis results mentioned above, it is indicated that the three tricyclic energetic compounds possess excellent molecular planarity and exhibit rich intermolecular interactions, which contribute to the enhancement of compound stability.

Due to the presence of hydrogen bond donor amino groups, in addition to intra-HBs, rich intermolecular hydrogen bonds (inter-HBs) were also observed in the crystals of the three compounds. Within **ATDT**·2H_2_O, one **ATDT** molecule forms three pairs of inter-HBs with four surrounding molecules, N(1)-H(1)⋯N(10), N(5)-H(5)⋯N(10), and N(8)-H(8B)⋯N(3) (1.916–2.575 Å), and one intramolecular N(8)-H(8B)⋯N(11) (2.231 Å). In the crystal of **ATNT**·2CH_3_OH, one **ATDT** molecule forms one pair of inter-HBs with two surrounding molecules, N(8)-H(8A)⋯N(4) (2.141 Å), and one intermolecular N(8)-H(8B)⋯N(9) (2.333 Å). Interestingly, the nitro groups do not engage in the formation of inter-HBs. In **ATDNP**·CH_3_OH, similar to **ATNT**·2CH_3_OH, as the orientation is opposite to the C3-NH_2_ group, C5–NO_2_ and C6–NO_2_ groups are also not involved in the formation of inter-HBs. One **ATDT** molecule forms three pairs of inter-HBs with three surrounding molecules: C3-H(6B)⋯N3, C3-H(6A)⋯N1, N2-H(2)⋯N5 (2.039–2.590 Å). The abundant intermolecular hydrogen bonds contribute to further enhancing the stability of tricyclic energetic compounds. More information about the bond lengths and the detail of crystal can be found in the Supporting Information.

### 2.3. Physicochemical and Energetic Properties

Table 1 summarizes the physicochemical and energy characteristics of three new compounds. An essential physical characteristic of EMs is their thermostatability, which is useful for determining how safe they are in harsh conditions. Using differential scanning calorimetry (DSC) at a heating rate of 5 °C min^−1^ in a dry nitrogen environment, the decomposition temperature (onset) was examined. During their breakdown, every compound showed distinct exothermic peaks (see Supporting Information). As shown in Table 1, all new compounds exhibit high decomposition temperatures, ranging from 264 to 361 °C. Both **ATNT** and **ATDNP** demonstrate high thermostability, with *T*_d_ values higher than 300 °C. Particularly noteworthy is the thermal decomposition temperature of **ATNT**, which exceeds 350 °C, surpassing the two typical heat-resistant explosives **HNS** (*T*_d_ = 318 °C) and **TATB** (*T*_d_ = 350 °C). Hence, they possess the potential to emerge as viable candidates for heat-resistant explosives.

Density is critical for energetic materials because it directly affects energy levels, such as detonation velocity (*vD*) and detonation pressure (*P*). Therefore, ensuring a high density has always been a primary objective. Utilizing a gas pyrometer set at 25 °C, densities were determined; the experimental densities of the three novel compounds ranged from 1.761 to 1.857 g cm^−3^. Two compounds have higher densities (**ATNT**: 1.836 g cm^−3^; **ATDNP**: 1.857 g cm^−3^) than **RDX** (1.80 g cm^−3^). Subsequently, the enthalpy of formation for all of the new energetic compounds were computed through the Gaussian 09 (Revision E.01) suite of programs [31]. Due to the high enthalpy of the major 1,2,4-triazole and tetrazole skeletons, **ATDT**, **ATNT**, and **ATDNP** show positive Δ*H*_f_ values as expected, ranging from 772.0 to 928.6 kJ mol^−1^, which are superior to those of **RDX** (74.8 kJ mol^−1^), **TATB** (−139.7 kJ mol^−1^), and **HNS** (−78.2 kJ mol^−1^). The results indicate that the tetrazole greatly enhances the heats of formation of the compounds. By utilizing EXPLO_5 (v 6.05.04) [32], the detonation velocity (*vD*) and pressure (*P*) of all new compounds were calculated based on existing data of density and Δ*H*_f_ values. From Table 1, the *vD* of the new materials falls between 8375 and 8502 m s^−1^, all exceeding “wood explosives” **TATB** (8179 m s^−1^) and **HNS** (7612 m s^−1^). Furthermore, the materials exhibit good detonation pressure (26.2–29.3 GPa). Apart from the energy level, the standard BAM methods [33] were used to assess the impact sensitivity (*IS*) and friction sensitivity (*FS*) of the three explosives. As shown in Table 1, the low mechanical sensitivities of **ATDT**, **ATNT**, and **ATDNP** (*IS* > 60 J, *FS* = 360 N) facilitate transportation and application. In fact, they benefit from a stable planar molecular skeleton and a regular packing model (cross and face-to-face stacking).

**Table 1:** Physicochemical and energetic properties of **ATDT**, **ATNT**, and **ATDNP**.

Comp	*T*_m_ ^a^ (°C)	*T*_d_ ^b^ (°C)	*d* ^c^ (g cm^−3^)	∆*H*_f_ ^d^ (kJ mol^−1^/kJ/g)	*vD* ^e^ (m s^−1^)	*P* ^f^ (GPa)	*IS* ^g^ (J)	*FS* ^h^ (N)
**ATDT**	229.8	264.4	1.761	928.59/(4.22)	8485	26.2	>60	360
**ATNT**	-	361.1	1.836	772.01/(2.92)	8375	26.8	>60	360
**ATDNP**	-	317.0	1.857	786.45/(2.55)	8502	29.3	>60	360
**RDX** ^i^	-	204	1.80	74.8/(0.25)	8762	33.6	7.4	120
**TATB** ^j^	-	350	1.93	−139.7/(−0.54)	8179	30.5	50	360
**HNS** ^k^	-	318	1.75	−78.2/(−0.17)	7612	24.3	5	240

^a^ Melt temperature. ^b^ Decomposition temperature (onset temperature at a heating rate of 5 °C min^−1^). ^c^ Density, measured-gas pycnometer (25 °C). ^d^ Calculated enthalpy of formation: Gaussian 09 (Revision E.01). ^e^ Calculated detonation velocity: EXPLO5 V6.05. ^f^ Calculated detonation pressure: EXPLO5 V6.05. ^g^ Impact sensitivity. ^h^ Friction sensitivity. ^i^ Ref. [34]. ^j^ Ref. [35]. ^k^ Ref. [36].

### 2.4. Theoretical Calculation Analysis

Understanding the factors influencing compound stability is crucial across various fields, including materials science and chemistry. The stability of energetic materials, which encompasses both its mechanical sensitivity and thermal stability, plays a vital role in their practical applications. To further elucidate the relationship between structure and performance, we employed various analytical techniques, including interaction region indicator (IRI) analysis [37], multicenter bond order, and localized orbital locator-π (LOL-π) [38] for crystals, as well as associated Hirshfeld surfaces and two-dimensional (2D) fingerprint analysis [39]. These methods provide insights into the stabilities of **ATDT**, **ATNT**, and **ATDNP** toward heat and external stimuli.

**Figure 3:**
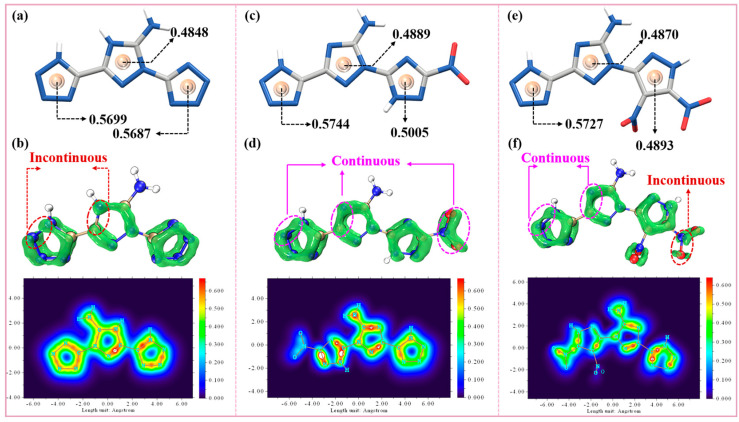
(**a**,**c**,**e**) The multicenter bond order analysis for **ATDT**, **ATNT**, and **ATDNP**; (**b**,**d**,**f**) the π−electron density distribution diagram of **ATDT**, **ATNT**, and **ATDNP** at the isosurface of 0.4 and the pathway of π−electron delocalization (1.2 bohr above the XY plane).

The aromaticity of energetic compounds significantly influences their thermostability. Therefore, we conducted a comparative analysis of aromaticity by evaluating the multicenter bond order and LOL-π of diverse heterocycles in the three compounds. For the three carbon-linked tetrazole rings, the multicenter bond order increases in the following order: **ATDT** (0.5699) < **ATDNP** (0.5727) < **ATNT** (0.5744). Similarly, for the 5-amino-1,2,4-triazole rings, the multicenter bond order increases in the following order: **ATDT** (0.4848) < **ATDNP** (0.4870) < **ATNT** (0.4889), indicating that the better aromaticity of the triazole and tetrazole rings in **ATNT** (Figure 3a,c,e). The LOL-π isosurface maps are depicted in Figure 3b,d,f. The distribution of π-electrons throughout the entire molecular framework and bonding nitro groups indicates good π-electron conjugation. However, there are discontinuities in the distribution of π–electrons in the tetrazole and triazole rings of **ATDT**, as well as in the nitro groups of **ATDNP**, while such discontinuities are absent in **ATNT**. This suggests that compound **ATNT** exhibits a higher π–electron delocalization threshold compared to the other compounds. This observation is consistent with the different thermal decomposition temperatures exhibited by the three compounds, as well as the experimental finding that compound **ATNT** possesses the highest thermal decomposition temperature.

**Figure 4:**
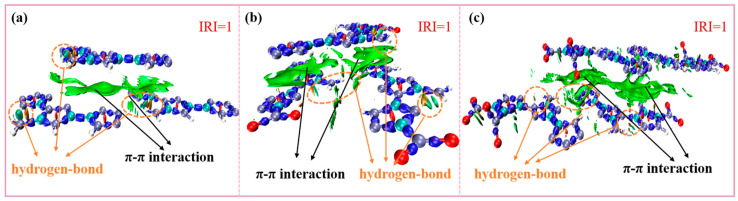
Isosurface map of chemicals (**a**) **ATDT**, (**b**) **ATNT**, and (**c**) **ATDNP** with IRI = 1.

Simultaneously, we employed IRI analysis to visually analyze of the weak intermolecular interactions during the crystal packing process. As illustrated in Figure 4, it is evident that there are abundant π–π and hydrogen bonding interactions in three compounds, depicted as large green isosurfaces. Notably, the distribution of π–π interactions in compounds **ATNT** and **ATDNP** appears more extensive than in **ATDT**, corroborating the higher thermal stability of compounds **ATNT** and **ATDNP**. The strategic arrangement of hydrogen bonds leads to the formation of good planar structures in the three compounds, fostering increased π–π interactions that significantly contribute to the materials’ overall stability under conditions of heat and various external stimuli.

**Figure 5:**
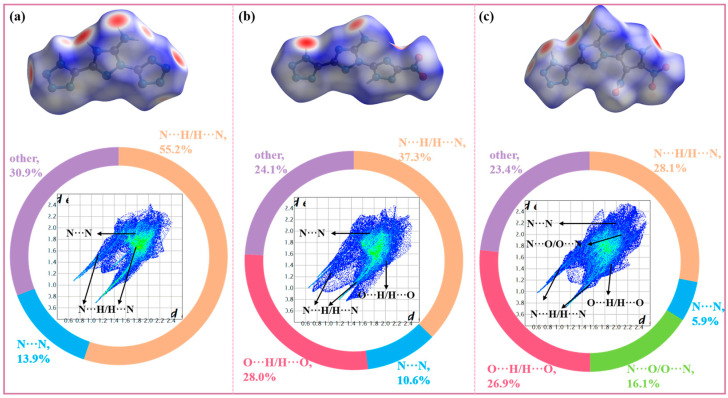
(**a**–**c**) the fingerprint plots, Hirshfeld surfaces, and percentage contribution for **ATDT**, **ATNT**, and **ATDNP**.

All three tricyclic energetic compounds exhibit characteristics of insensitivity. Analysis of Hirshfeld surfaces and 2D fingerprints (Figure 5) reveals nearly planar conformations in the Hirshfeld surfaces of the three new compounds. Furthermore, based on 2D fingerprint plots, it is evident that all three compounds exhibit abundant, weak interactions. In the case of **ATDT**, the major interactions are N⋯H/H⋯N (55.2%) and N⋯N (13.9%). Despite the presence of nitro groups in **ATNT** and **ATDNP**, the primary interactions still arise from N⋯H/H⋯N (37.3% and 28.1%, respectively), rather than O⋯H/H⋯O (28.0% and 26.9%, respectively), due to the opposing orientation of the nitro and amino groups. This further confirms the efficacy of introducing amino groups into the central 1,2,4-triazole ring in the design.

## 3. Materials and Methods

### 3.1. General Methods

^1^H and ^13^C NMR spectra were determined using a Bruker 400MHz spectrometer (400 and 100 MHz, respectively) in d-DMSO. Chemical shifts are reported as δ values relative to the internal standard d-DMSO (δ 2.50 for ^1^H NMR and 39.52 for ^13^C NMR). Infrared spectra (IR) were obtained on a PerkinElmer Spectrum BX FT-IR instrument equipped with an ATR unit at 25 °C. Elemental analyses of C/H/N were carried out using a Vario EL III Analyzer. The onset decomposition temperature was measured using a TA Instruments DSC25 differential scanning calorimeter at a heating rate of 5 °C min^−1^ in a dry nitrogen atmosphere. Impact and friction sensitivities were measured with a BAM Fallhammer and friction tester. Densities were calculated at 296 K based on crystal structure data and measured by Micromeritics AccuPyc 1345 gas pycnometer. X-ray diffractions of all single crystals were carried out on a Bruker D8 VENTURE diffractometer using Mo-Kα radiation (λ = 0.71073 Å). The data collection was performed using the APEX III software package67 on single crystals coated with Fomblin Y as perfluoropolyether. The single crystals were selected on a MiTiGen MicroMount microsampler, transferred to the diffractometer, and measured. The heats of formation for complex structures were obtained by atomization using G2 ab initio method [40,41]. The enthalpy of sublimation was calculated by using Trouton’s rule [42]. Additional details for data processing, structure refinement, and graphic depictions are given in the supplementary information.

The samples were dried under reduced pressure vacuum for at least 12 h to ensure that they are free from organic solvents and water, thus avoiding any potential impact on the test results. Additionally, all samples to be tested underwent elemental analysis to ensure their purity.

### 3.2. Synthetic Procedures

Potassium 5-amino-1-(1H-tetrazol-5-yl)-1H-1,2,4-triazole-3-carbonitrile (**1**)*:* At 0 °C, 60 mL of anhydrous acetonitrile was used to dissolve 10 mmol of cyanogen bromide. Then, 40 mmol of sodium azide was carefully added. After stirring the mixture for five hours at a temperature within 0 and 5 °C, the inorganic salt was filtered out. At 0 °C, 20 mL of water was used to dissolve 5 mmol of potassium 5-amino-1H-1,2,4-triazole-3-carbonitrile (**ACT-K**). The cyanogen azide solution was then slowly added. The solvent was eliminated by air after the mixture was stirred for seven hours at room temperature, leading to a solid precipitate that was filtered and then cleaned with acetonitrile and water. Yield 60%, yellow solid. ^1^H NMR (400 MHz, *d*_6_-DMSO): δ 7.95 (s, 2H). ^13^C NMR (101 MHz, *d*_6_-DMSO): δ 113.3, 135.9, 155.4, 157.6. IR (KBr pellet): ṽ 3301, 3235, 3162, 2258, 1645, 1571, 1537, 1481, 1429, 1407, 1342, 1211, 1155, 1127, 1092, 1059, 1038, 1016, 1008, 801, 742, 697, 618, 577, 485 cm^−1^. Elemental analysis for C_4_H_2_KN_9_ (215.0): Calcd C 22.32, H 0.94, N 58.57. Found: C 22.28, H 1.03, N 59.01.

5-Amino-5′-nitro-2′H-[1,3′-bi(1,2,4-triazole)]-3-carbonitrile (**2**)*:* In 5 mL of methanol, potassium of 5-amino-1H-1,2,4-triazole-3-carbonitrile (**ACT-K**) (10 mmol) was dissolved. The methanol solution was then supplemented with K_2_CO_3_ (10 mmol) and 1,3-dinitro-triazole (10 mmol) in methanol (20 mL), respectively. After six hours of stirring at 60 °C, the substrate was eliminated from the mixture, and the MeOH was subsequently extracted under reduced pressure. After dissolving the remaining material in 50 mL of water, diluted hydrochloric acid was added to the resulting solution to make it acidic. After filtering, icy water was used to wash the yellow precipitate. Yield 78%, yellow solid. ^1^H NMR (400 MHz, *d*_6_-DMSO): δ 7.80 (s, 2H). ^13^C NMR (101 MHz, *d*_6_-DMSO): δ 112.3, 137.9, 148.4, 155.8, 160.4. IR (KBr pellet): ṽ 3575, 3389, 3310, 2428, 2259, 1643, 1602, 1566, 1547, 1498, 1469, 1404, 1371, 1305, 1184, 1137, 1029, 970, 840, 788, 738, 713, 642, 501, 475 cm^−1^. Elemental analysis for C_5_H_3_N_9_O_2_ (221.0): Calcd C 27.16, H 1.37, N 57.01. Found: C 27.13, H 1.46, N 56.56.

5-Amino-1-(3,4-dinitro-1H-pyrazol-5-yl)-1H-1,2,4-triazole-3-carbonitrile (**3**)*:* In 5 mL of methanol, potassium of 5-amino-1H-1,2,4-triazole-3-carbonitrile (**ACT-K**) (10 mmol) was dissolved. The methanol solution was then supplemented with K_2_CO_3_ (10 mmol) and 1,3,4-trinitropyrazole (10 mmol) in methanol (20 mL), respectively. After six hours of stirring at 60 °C, the substrate was eliminated from the mixture, and the MeOH was subsequently extracted under low pressure. After dissolving the remaining material in 50 mL of water, diluted hydrochloric acid was added to the resulting solution to make it acidic. The yellow precipitate was filtered and washed with ice water. Yield 78%, yellow solid. ^1^H NMR (400 MHz, *d*_6_-DMSO): δ 7.17 (s, 2H). ^13^C NMR (101 MHz, *d*_6_-DMSO): δ 113.1, 119.4, 136.4, 138.2, 150.6, 157.4. IR (KBr pellet): ṽ 3417, 3303, 3100, 2949, 2266, 1644, 1529, 1488, 1390, 1339, 1244, 1181, 1089, 1029, 974, 927, 841, 806, 748, 735, 706, 625, 572, 522, 440 cm^−1^. Elemental analysis for C_6_H_3_N_9_O_4_ (265.0): Calcd C 27.18, H 1.14, N 47.54. Found: C 26.99, H 1.18, N 46.56.

1,3-Di(1H-tetrazol-5-yl)-1H-1,2,4-triazol-5-amine (**ATDT**), 5′-nitro-3-(1H-tetrazol-5-yl)-2′H-[1,3′-bi(1,2,4-triazol)]-5-amine (**ATNT**), and 1-(3,4-dinitro-1H-pyrazol-5-yl)-3-(1H-tetrazol-5-yl)-1H-1,2,4-triazol-5-amine (**ATDNP**)*:* Potassium 5-amino-1-(1H-tetrazol-5-yl)-1H-1,2,4-triazole-3-carbonitrile (**1**), 5-amino-5′-nitro-2′H-[1,3′-bi(1,2,4-triazole)]-3-carbonitrile (**2**) and 5-amino-1-(3,4-dinitro-1H-pyrazol-5-yl)-1H-1,2,4-triazole-3-carbonitrile (**3**) were dissolved in 15 mL water, respectively. After that, the mixture was refluxed for two hours while zinc chloride (6 mmol) and sodium azide (6 mmol) were added gradually. To stop zinc hydroxide from precipitating, 5 mL of a 10% HCl solution was added once the mixture had cooled to room temperature. The precipitate was air-dried after being filtered and washed with ice water.

1,3-Di(1H-tetrazol-5-yl)-1H-1,2,4-triazol-5-amine (**ATDT**): Yield: 94%, colorless solid. ^1^H NMR (400 MHz, *d*_6_-DMSO): δ 7.66 (s, 2H). ^13^C NMR (101 MHz, *d*_6_-DMSO): δ 149.4, 150.4, 153.7, 156.7. IR (KBr pellet): ṽ 3531, 3374, 3141, 2444, 1892, 1652, 1575, 1502, 1479, 1402, 1271, 1228, 1172, 1148, 1028, 1008, 957, 742, 730, 687, 648, 575, 512, 452, 419 cm^−1^. HRMS (ESI) *m*/*z*: 219.06020 (M–H)^−^. Elemental analysis for C_4_H_4_N_12_ (220.0): Calcd C 21.82, H 1.83, N 76.35. Found: C 21.65, H 1.90, N 76.41.

5′-Nitro-3-(1H-tetrazol-5-yl)-2′H-[1,3′-bi(1,2,4-triazol)]-5-amine (**ATNT**): Yield: 95%, yellow solid. ^1^H NMR (400 MHz, *d*_6_-DMSO): δ 7.48 (s, 2H). ^13^C NMR (101 MHz, *d*_6_-DMSO): δ 148.2, 152.2, 153.7, 154.6, 164.2. IR (KBr pellet): ṽ 3427, 1654, 1633, 1549, 1521, 1508, 1424, 1322, 1309, 1211, 1141, 1077, 1021, 989, 841, 792, 732, 679, 641, 480, 438 cm^−1^. HRMS (ESI) *m*/*z*: 263.05032 (M–H)^−^. Elemental analysis for C_5_H_4_N_12_O_2_ (264.0): Calcd C 22.73, H 1.53, N 63.63. Found: C 22.89, H 1.68, N 62.53.

1-(3,4-Dinitro-1H-pyrazol-5-yl)-3-(1H-tetrazol-5-yl)-1H-1,2,4-triazol-5-amine (**ATDNP**): Yield: 90%, yellow solid. ^1^H NMR (400 MHz, *d*_6_-DMSO): δ 6.35 (s, 2H). ^13^C NMR (101 MHz, *d*_6_-DMSO): δ 120.4, 136.2, 148.9, 149.2, 149.3, 157.9. IR (KBr pellet): ṽ 3572, 3451, 3345, 2323, 1849, 1645, 1569, 1543, 1517, 1401, 1359, 1313, 1275, 1244, 1230, 1147, 1122, 1103, 1039, 968, 844, 812, 763, 730, 705, 614, 413 cm^−1^. HRMS (ESI) *m/z*: 307.04031 (M–H)^−^. Elemental analysis for C_6_H_4_N_12_O_4_ (308.0): Calcd C 23.38, H 1.31, N 54.54. Found: C 22.79, H 1.53, N 54.58.

## 4. Conclusions

In this work, we introduced amino groups into the central ring of tricyclic energetic compounds to construct intramolecular hydrogen bonds. Three new tricyclic energetic compounds were synthesized with satisfactory skeleton planarity. They exhibit insensitivity (*IS* > 60 J; *FS* = 360 N) when exposed to external mechanical stimuli. Notably, **ATNT** and **ATDNP** demonstrate high decomposition temperature (361.1 °C and 317.0 °C, respectively). Furthermore, they exhibit better detonation performances (**ATNT**, *vD*: 8375 m s^−1^; **ATDNP**, *vD*: 8502 m s^−1^). Consequently, **ATNT** and **ATDNP** have significant application potential as candidate heat-resistant explosives. Constructing tricyclic frameworks and enhancing molecular planarity by designing intramolecular hydrogen bonds are effective strategies for preparing high-safety energetic materials. This approach achieves a larger conjugation area and enhances π–π interactions. These results may inspire new thinking with regard to the construction of new tricyclic energetic compounds with good planarity, holding promise for practical applications.

## Data Availability

Data are contained within the article and Supplementary Materials.

## Supplementary Materials

The following supporting information can be downloaded at: https://www.mdpi.com/article/10.3390/ijms25073910/s1.
