# Supplementary material for: Intramolecular Hydrogen Bonds Assisted Construction of Planar Tricyclic Structures for Insensitive and Highly Thermostable Energetic Materials
Source: Int J Mol Sci. 2024 Mar 31;25(7):3910. doi: 10.3390/ijms25073910 (PMC11012039; doi:10.3390/ijms25073910)
Supplement: Supplementary file 1 [file ijms-25-03910-s001.zip › ijms-2922431-supplementary.pdf]

Supporting Information (SI)

Table of Contents

1. Theoretical calculation..2

2. X-ray Crystallographic Datas 3

3. Spectrums for all new compounds...6

4. HRMS spectrum for ATDT, ATNT, ATDNP...12

5. IR spectra of all new compounds...13

6. DSC plots for ATDT, ATNT and ATDNP 15

References..17

1. Theoretical calculation

The calculation of the heats of formation were carried out using Gaussian 09 (Revision E.01) suite of programs. All the compounds were determined using isodesmic reactions (Scheme S1). The geometric optimization and frequency analyses of the structures were calculated using B3LYP/6-311++G** level, and the single energy points were calculated at the M062X/de2tzvpp level. The heats of formation for complex structures were obtained by atomization using G2 ab initio method [1]. The enthalpy of sublimation was calculated by using Trouton's rule [2]. Solid state heats of formation of the resulting compounds were calculated with equation (1) in which $T_{m/d}$ is the melting temperature or decomposition temperature.

$$\Delta H_{f(solid)} = \Delta H_{f(g)} - \Delta H_{sub} = \Delta H_{f(g)} - 188[J\ mol^{-1}\ K^{-1}] * T_{m/d} \quad (1)$$

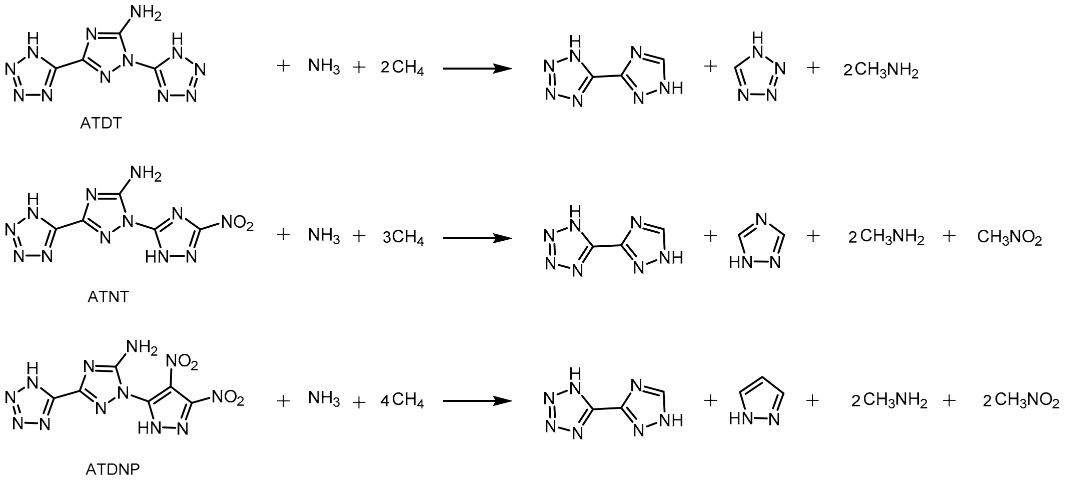

Scheme S1. Isodesmic reactions.

Table S1. Zero-point energy (ZPE), values of the correction (H_{corr}), calculated total energy (E_0), enthalpy of formation in gas-state (ΔH_{gas}), lattice energy (ΔH_L), enthalpy of sublimation (ΔH_{sub}) and enthalpy of formation in solid-state (ΔH_{solid}) for compounds **ATDT**, **ATNT** and **ATDNP**.

**Comp.**	**ZPE (a.u.)**	**H_{corr} (a.u.)**	**E_0 (a.u.)**	**Corrected E_0**	**$\Delta_f H_{gas}$ (kJ mol^{-1})**	**$\Delta_f H_{sub}$ (kJ mol^{-1})**	**$\Delta_f H_{solid}$ (kJ mol^{-1})**
CH_4	0.044599	0.048412	-40.5018	-40.45518	-74.60 [a]		
NH_3	0.034304	0.038113	-56.5519	-56.5152	-45.90 [a]		
CH_3NH_3	0.063823	0.068180	-95.8452	-95.77960	-23.50 [a]		
CH_3NO_2	0.049691	0.055014	-245.0103	-244.9573	-81.00 [a]		
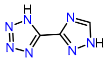	0.087381	0.095236	-499.3280	-499.2363	568.52		

	0.046864	0.051271	-258.2556	-258.2062	335.14		
	0.059735	0.064226	-242.2515	-242.1897	194.04		
	0.071101	0.075779	-226.1952	-226.1222	183.66		
**ATDT**	0.130858	0.143739	-811.7473	-811.6088	972.59	44.00	928.59
**ATNT**	0.145809	0.161416	-1000.2438	-1000.0882	839.88	67.87	772.01
**ATDNP**	0.159903	0.178404	-1188.6857	-1188.5137	846.04	59.59	786.45

[a] Data from NIST WebBook.

2. X-ray Crystallographic Datas

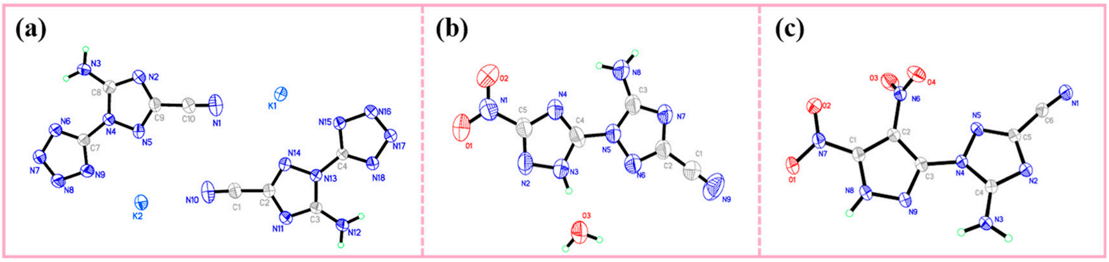

Figure S1. Crystal structures of compounds (a) **1**, (b) **2**·$2H_2O$ and (c) **3**.

Table S2. Crystal data, data collection, and refinement for **ATDT**, **ATNT** and **ATDNP**

	**ATDT**·$2H_2O$	**ATNT**·$2CH_3OH$	**ATDNP**·CH_3OH
CCDC No.	2201504	2233195	2233194
Empirical Formula	$C_4H_8N_{12}O_2$	$C_7H_{12}N_{12}O_4$	$C_7H_7N_{12}O_5$
Formula Weight	256.22	328.29	339.25
Temperature (K)	296(2)	298	298
Crystal System	monoclinic	monoclinic	monoclinic
Space group	Cc	$P2_1/n$	$P2_1/c$
a (Å)	4.880(3)	14.3044(16)	9.2902(6)
b (Å)	13.318(8)	7.2539(6)	7.3946(5)
c (Å)	15.925(9)	15.4953(19)	20.0556(13)
α (°)	90	90	90
β (°)	98.420(8)	116.576(3)	101.870(2)
γ (°)	90	90	90
Volume ($Å^3$)	1023.9(10)	1438.0(3)	1348.30(15)
Z	4	4	4

Density ($g \cdot cm^{-3}$)	1.662	1.516	1.671
μ (mm^{-1})	0.137	0.126	0.143
F (000)	528.0	680.0	692.0
Crystal size/mm^3	0.18 × 0.15 × 0.08	0.15 × 0.08 × 0.05	0.12 × 0.08 × 0.05
Index ranges	$-6 \leq h \leq 6$<br>$-17 \leq k \leq 15$<br>$-20 \leq l \leq 19$	$-17 \leq h \leq 16$<br>$-8 \leq k \leq 9$<br>$-16 \leq l \leq 19$	$-11 \leq h \leq 11$<br>$-9 \leq k \leq 8$<br>$-24 \leq l \leq 24$
2Θ range for data collection (°)	5.172 to 55.558	5.212 to 52.828	4.15 to 52.316
Reflections collected	3174	9973	14439
Independent reflections	1988[R_{int} = 0.0455, R_{sigma} = 0.0794]	2876[R_{int} = 0.0763, R_{sigma} = 0.0772]	2692[R_{int} = 0.0569, R_{sigma} = 0.0424]
Data/restraints/parameters	1988/8/179	2876/162/228	2962/0/219
Goodness-of-fit on F^2	1.100	1.078	1.064
Final R indexes [I>=2σ(I)]	R_1= 0.0541, wR_2 = 0.0983	R_1= 0.0642, wR_2 = 0.1400	R_1= 0.0545, wR_2 = 0.1264
Final R indexes [all data]	R_1 =0.0734, wR_2 = 0.1059	R_1 = 0.1270, wR_2 = 0.1788	R_1= 0.0933, wR_2 = 0.1543
Largest diff. peak/hole ($e \cdot Å^{-3}$)	0.28/-0.28	0.43/-0.34	0.65/-0.30

Table S3. Crystal data, data collection, and refinement for **1**, **2**$\cdot H_2O$ and **3**.

	**1**	**2**$\cdot H_2O$	**3**
CCDC No.	2233193	2244941	2233192
Empirical Formula	$C_8H_4K_2N_{18}$	$C_5H_5N_9O_3$	$C_6H_3N_9O_4$
Formula Weight	430.49	239.18	265.17
Temperature (K)	296(2)	298	170
Crystal System	monoclinic	monoclinic	monoclinic
Space group	$P2_1/c$	$P2_1/n$	$P2_1/n$
a (Å)	15.9919(9)	14.321(14)	5.8589(7)
b (Å)	7.7897(4)	4.065(4)	13.1608(18)
c (Å)	12.8828(7)	17.180(18)	12.4118(15)
α (°)	90	90	90
β (°)	98.944(2)	99.80(5)	90.115(3)
γ (°)	90	90	90
Volume ($Å^3$)	1585.32(15)	985.4(17)	957.0(2)

Z	4	4	4
Density (g·cm^{-3})	1.804	1.612	1.840
μ (mm^{-1})	0.643	0.136	0.158
F (000)	864	488	536.0
Crystal size/mm^3	0.20 × 0.20 × 0.20	0.12 × 0.04 × 0.02	0.07 × 0.05 × 0.04
Index ranges	$-19 \le h \le 16$<br>$-8 \le k \le 9$<br>$-14 \le l \le 15$	$-16 \le h \le 17$<br>$-5 \le k \le 5$<br>$-21 \le l \le 21$	$-7 \le h \le 7$<br>$-16 \le k \le 16$<br>$-15 \le l \le 14$
2Θ range for data collection (°)	1.289 to 25.003	4.06 to 53.078	5.212 to 52.828
Reflections collected	23340	6539	7614
Independent reflections	2783[R_{int} = 0.0555	1925[R_{int} = 0.0582, R_{sigma} = 0.0552]	1960[R_{int} = 0.0547, R_{sigma} = 0.0492]
Data/restraints/parameters	2783/0/253	1925/1/161	1960/3/184
Goodness-of-fit on F^2	1.039	1.042	1.076
Final R indexes [I>=2σ(I)]	R_1= 0.0400, wR_2 = 0.0909	R_1= 0.0818, wR_2 = 0.2174	R_1= 0.0437, wR_2 = 0.1013
Final R indexes [all data]	R_1 =0.0738, wR_2 = 0.1067	R_1 = 0.1344, wR_2 = 0.2627	R_1 = 0.0704, wR_2 = 0.1216
Largest diff. peak/hole (e·Å^{-3})	0.232/-0.258	0.60/-0.28	0.21/-0.28

Table S4. Bond Length in Å for **ATDT**·$2H_2O$.

**Atom1**	**Atom1**	Length/Å	**Atom1**	**Atom1**	Length/Å
N1	N2	1.347(6)	N7	C1	1.397(5)
N1	C4	1.312(6)	N7	C2	1.351(5)
N2	N3	1.304(6)	N8	C2	1.322(6)
N3	N4	1.358(6)	N9	N10	1.328(6)
N4	C4	1.331(6)	N9	C1	1.321(6)
N5	C2	1.311(6)	N10	N12	1.314(6)
N5	C3	1.356(6)	N11	N12	1.326(5)
N6	N7	1.390(6)	N11	C1	1.335(6)
N6	C3	1.310(6)	C3	C4	1.454(6)

Table S5. Bond Length in Å for **ATNT**·$2CH_3OH$.

**Atom1**	**Atom1**	Length/Å	**Atom1**	**Atom1**	Length/Å
N6	N5	1.389(3)	O1	C6	1.400(4)

N6	C3	1.378(4)	N4	N3	1.356(4)
N6	C4	1.365(4)	N4	C1	1.325(4)
N7	C2	1.362(4)	N3	N2	1.305(4)
N7	C3	1.334(4)	N8	C3	1.327(4)
N5	C2	1.315(4)	N9	C4	1.321(4)
N1	N2	1.344(3)	N9	C5	1.348(4)
N1	C1	1.325(4)	N10	C4	1.331(4)

Table S6. Bond Length in Å for **ATDNP**·CH_3OH.

**Atom1**	**Atom1**	Length/Å	**Atom1**	**Atom1**	Length/Å
O5	C7	1.425(4)	N10	C6	1.335(4)
N7	N8	1.381(3)	N2	N1	1.335(3)
N7	C4	1.397(3)	N2	C1	1.322(3)
N7	C3	1.362(3)	N4	N3	1.364(3)
N5	C3	1.334(3)	N4	C1	1.324(3)
N5	C2	1.360(3)	O3	N11	1.229(3)
N9	N10	1.351(3)	N12	O1	1.209(3)
N9	C4	1.328(3)	N12	C5	1.451(4)
N8	C2	1.314(3)	O4	N11	1.209(3)
O2	N12	1.211(3)			

3. Spectrums for all new compounds

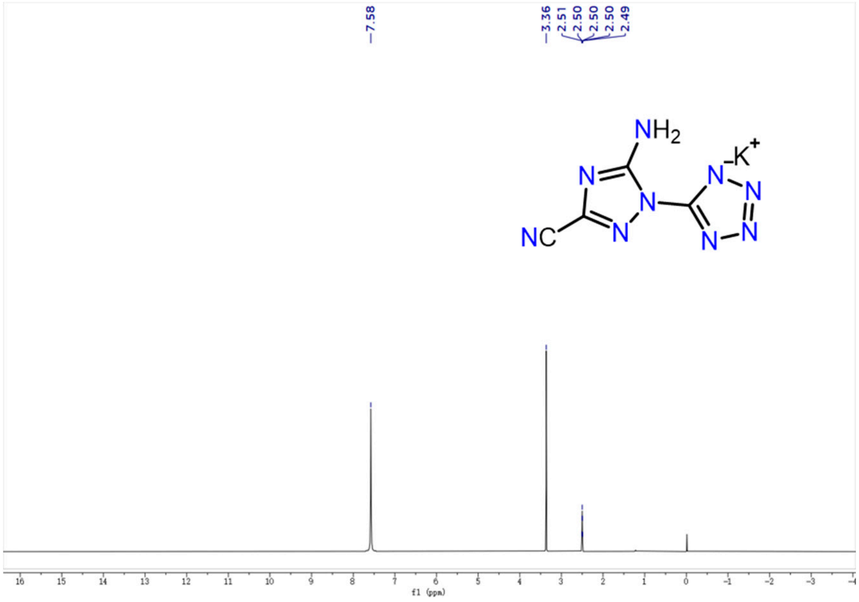

Figure S2. ^{1}H NMR spectrum of **1** in d_6-DMSO.

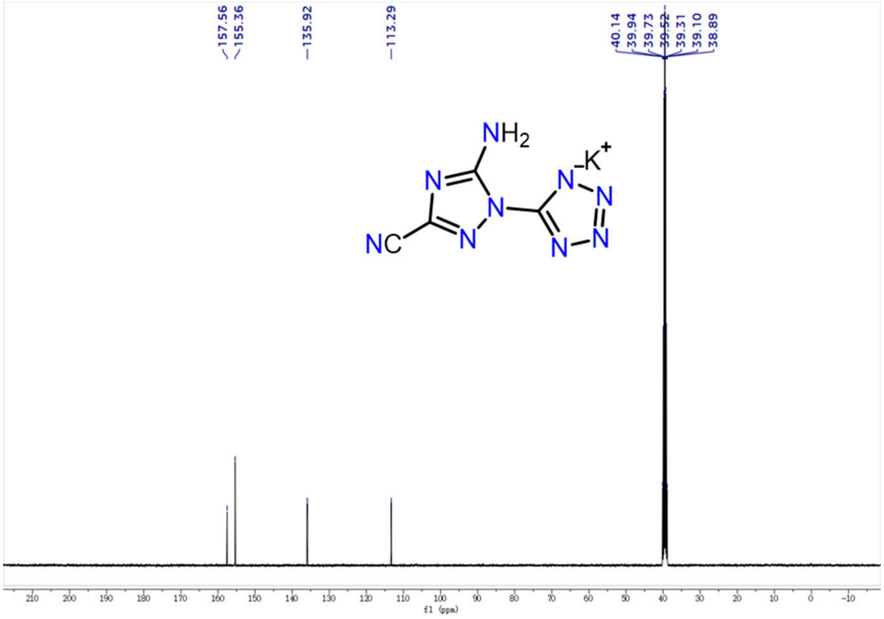

Figure S3. ^{13}C NMR spectrum of **1** in d_6-DMSO.

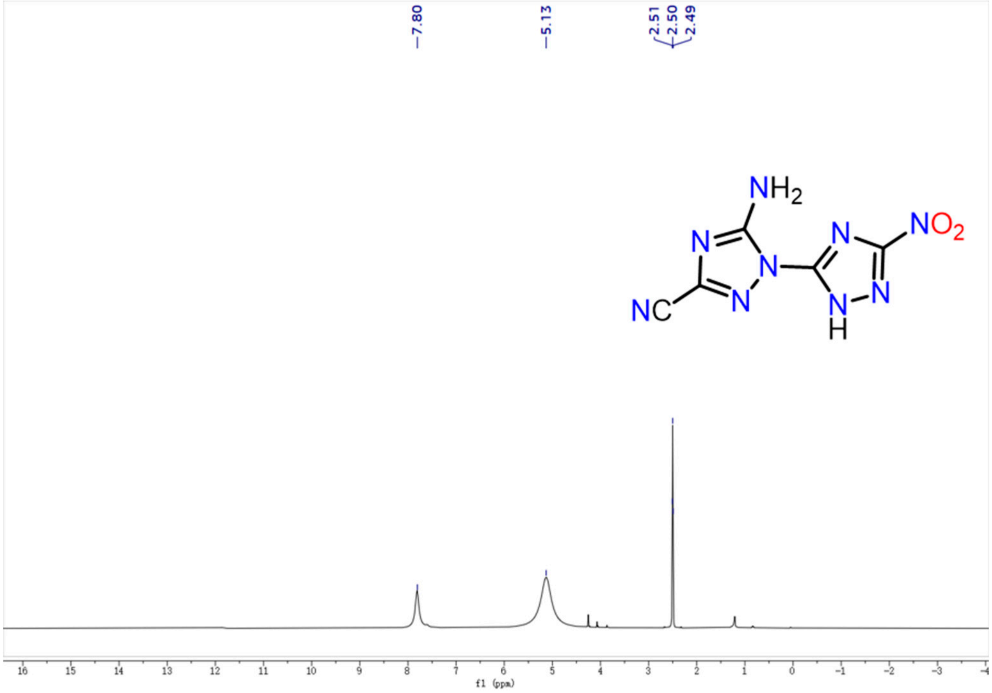

Figure S4. ^{1}H NMR spectrum of **2** in d_6-DMSO.

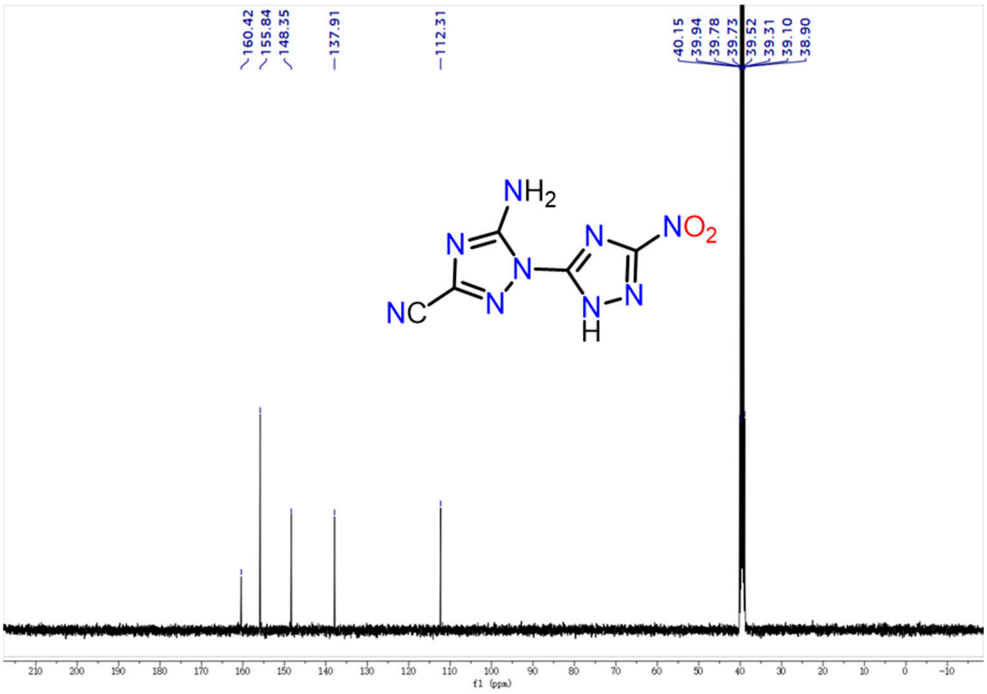

Figure S5. ^{13}C NMR spectrum of **2** in d_6-DMSO.

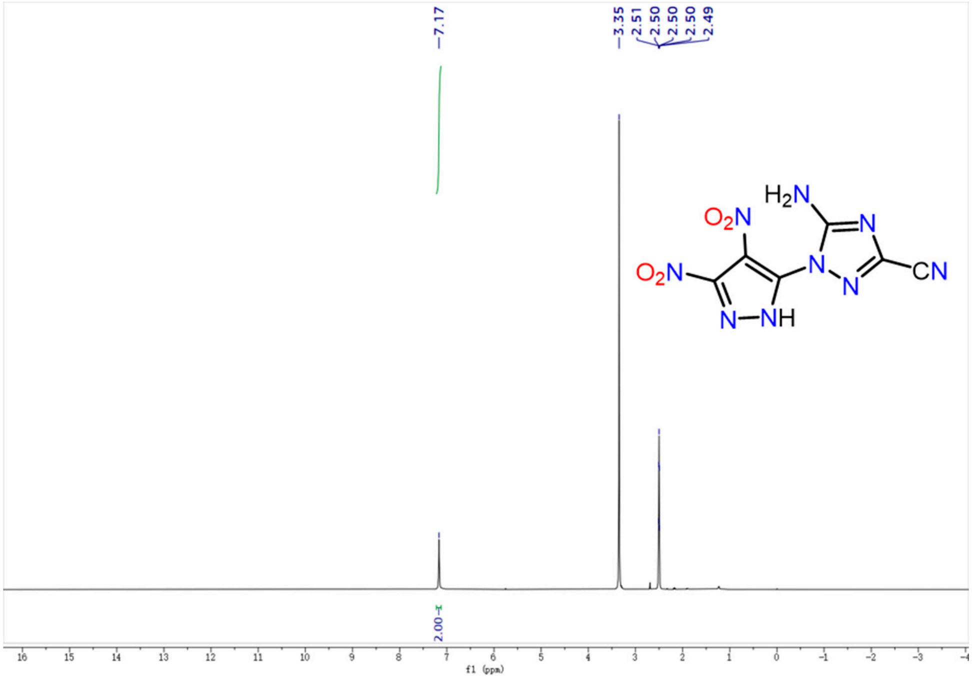

Figure S6. ^{1}H NMR spectrum of **3** in d_6-DMSO.

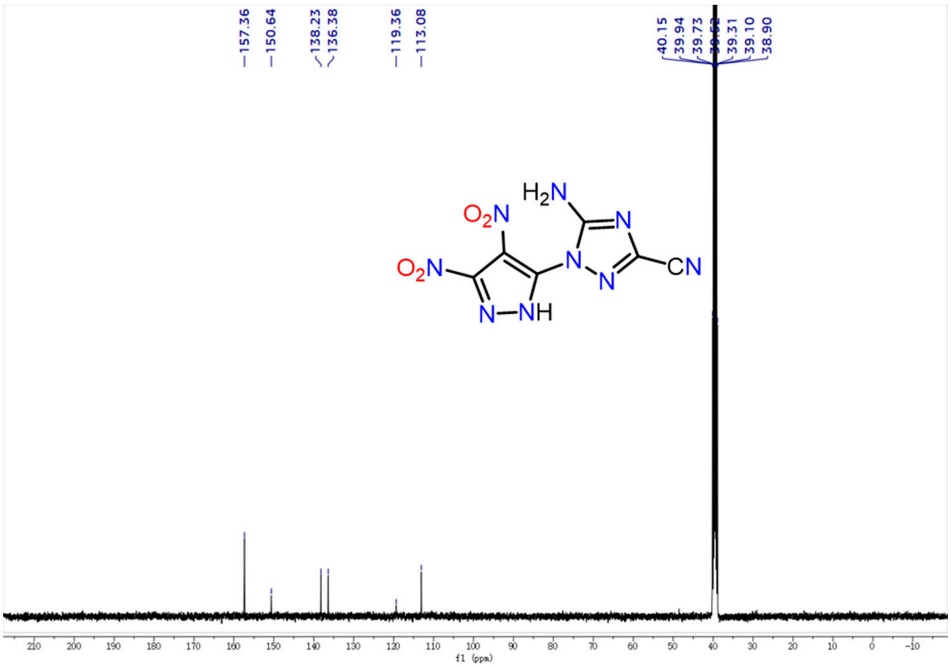

Figure S7. ^{13}C NMR spectrum of **3** in d_6-DMSO.

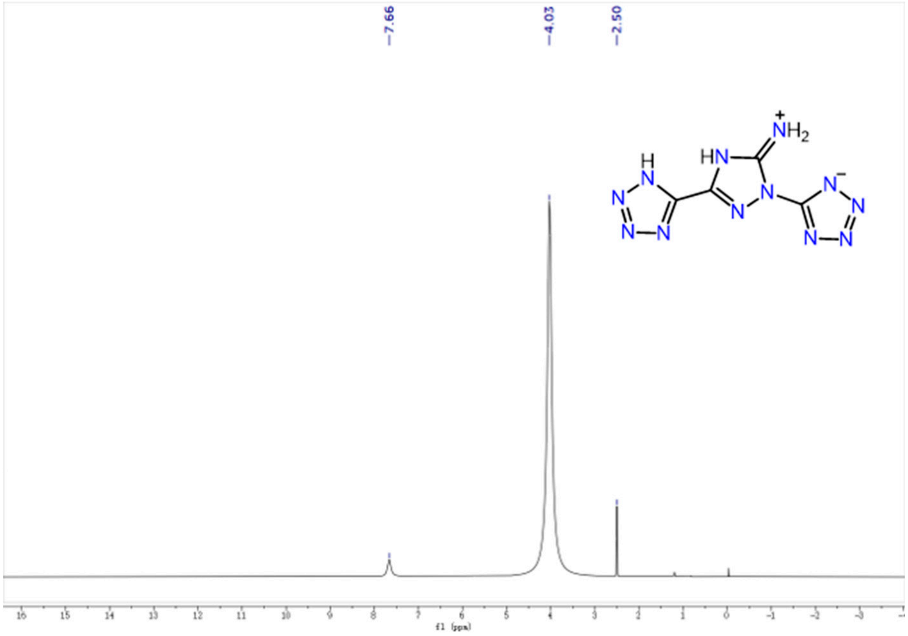

Figure S8. ^{1}H NMR spectrum of **ATDT** in d_6-DMSO.

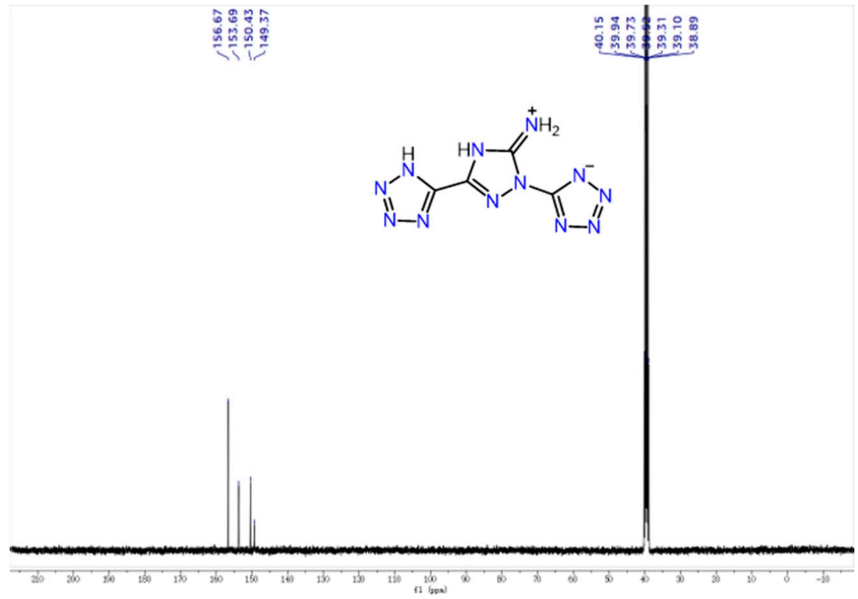

Figure S9. ^{13}C NMR spectrum of **ATDT** in d_6-DMSO.

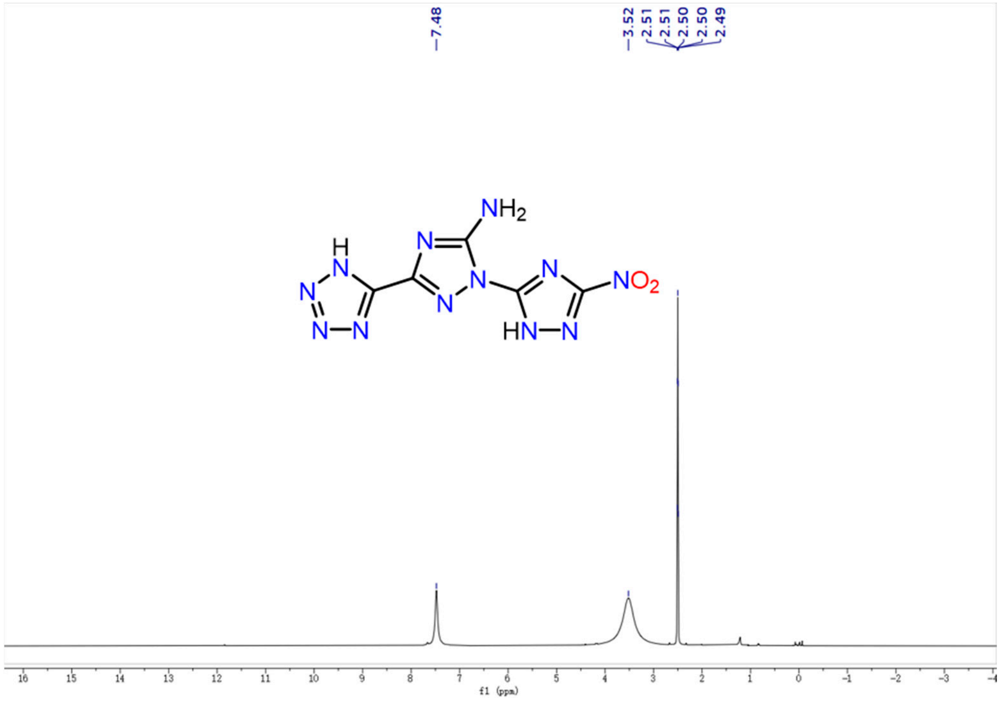

Figure S10. ^{1}H NMR spectrum of **ATNT** in d_6-DMSO.

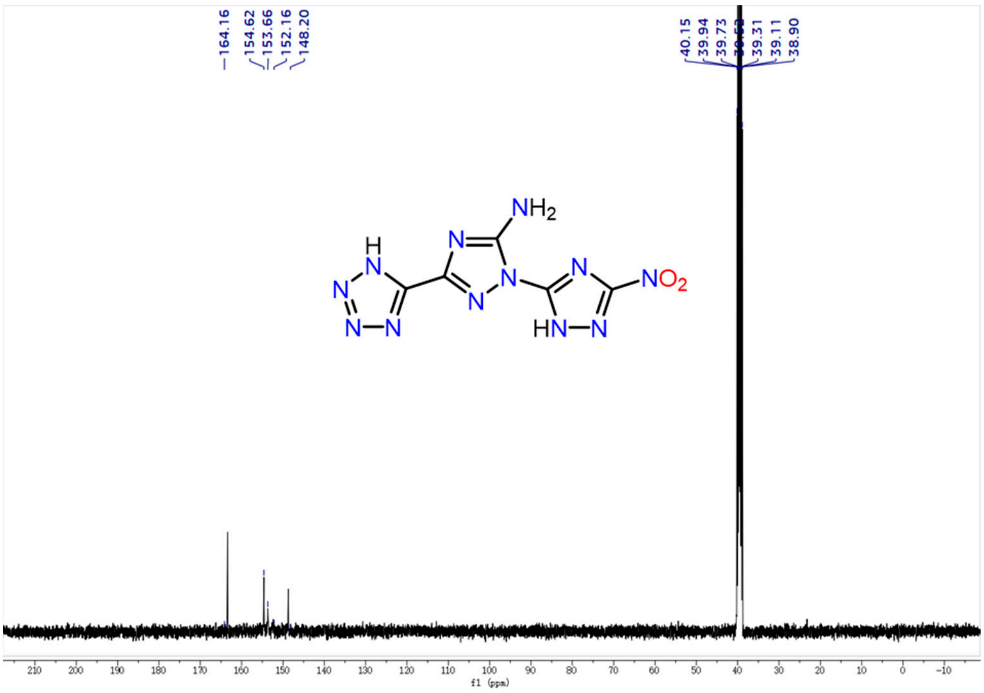

Figure S11. ^{13}C NMR spectrum of **ATNT** in d_6-DMSO.

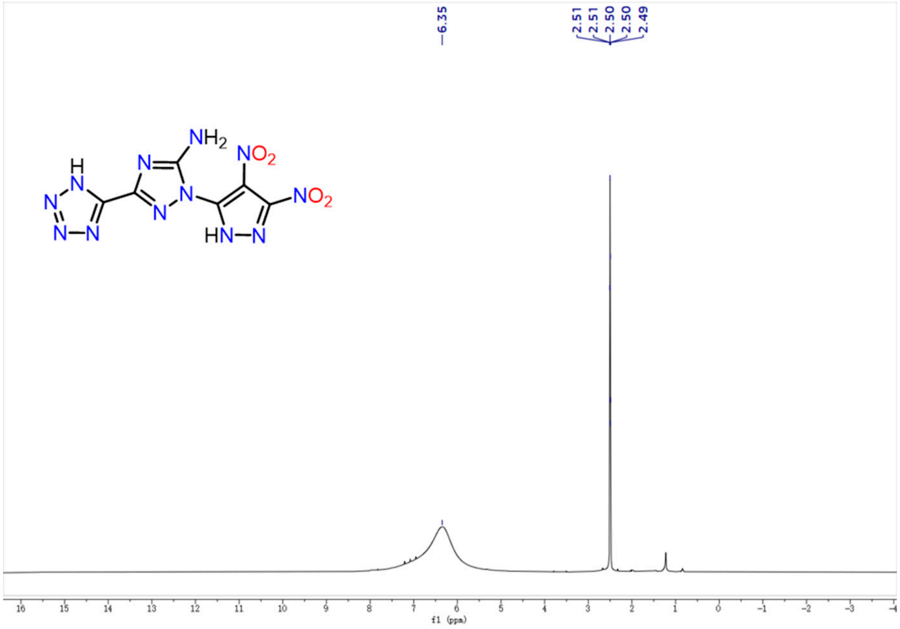

Figure S12. ^{1}H NMR spectrum of **ATDNP** in d_6-DMSO.

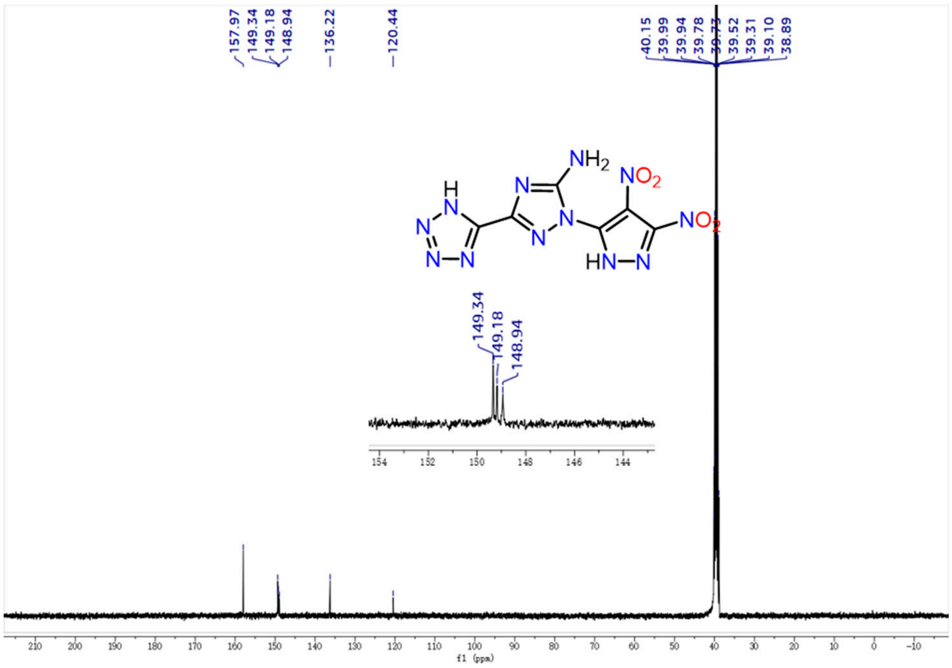

Figure S13. ^{13}C NMR spectrum of **ATDNP** in d_6-DMSO.

4. HRMS spectrum for ATDT, ATNT, ATDNP

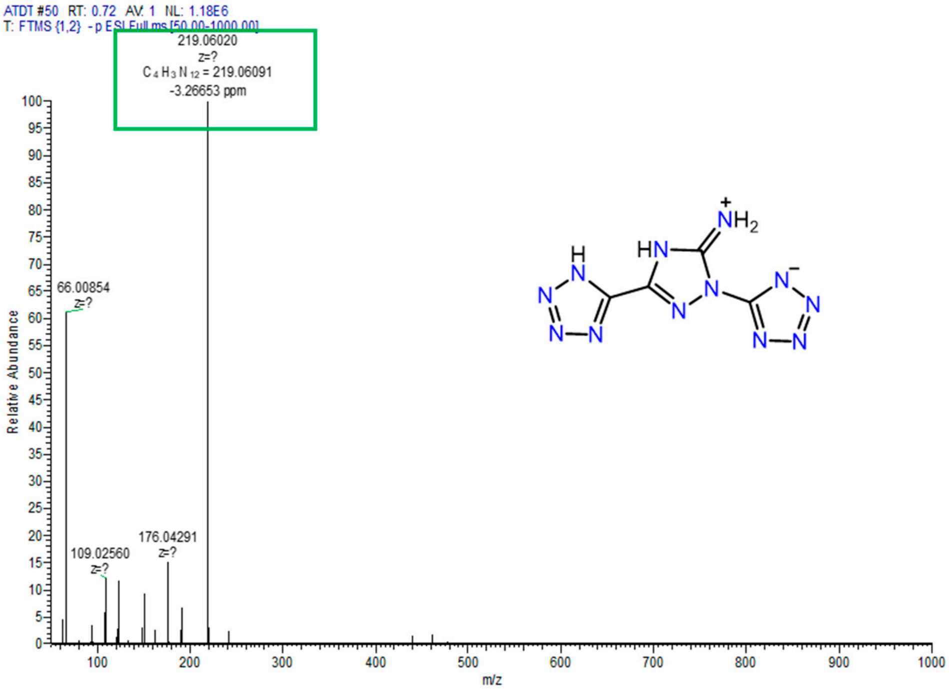

Figure S14. HRMS spectrum for **ATDT**

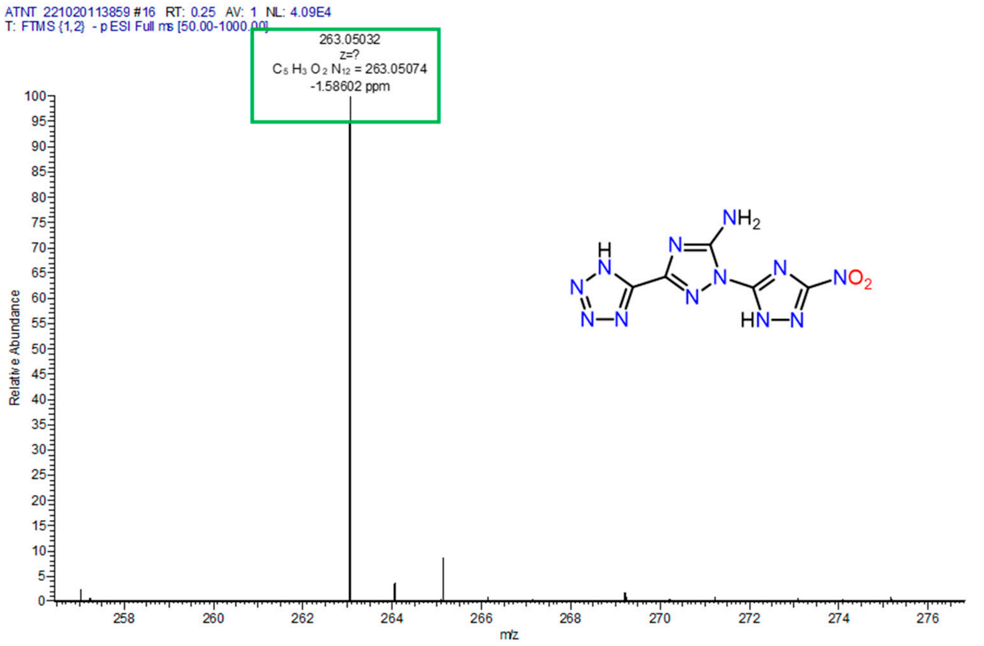

Figure S15. HRMS spectrum for **ATNT**

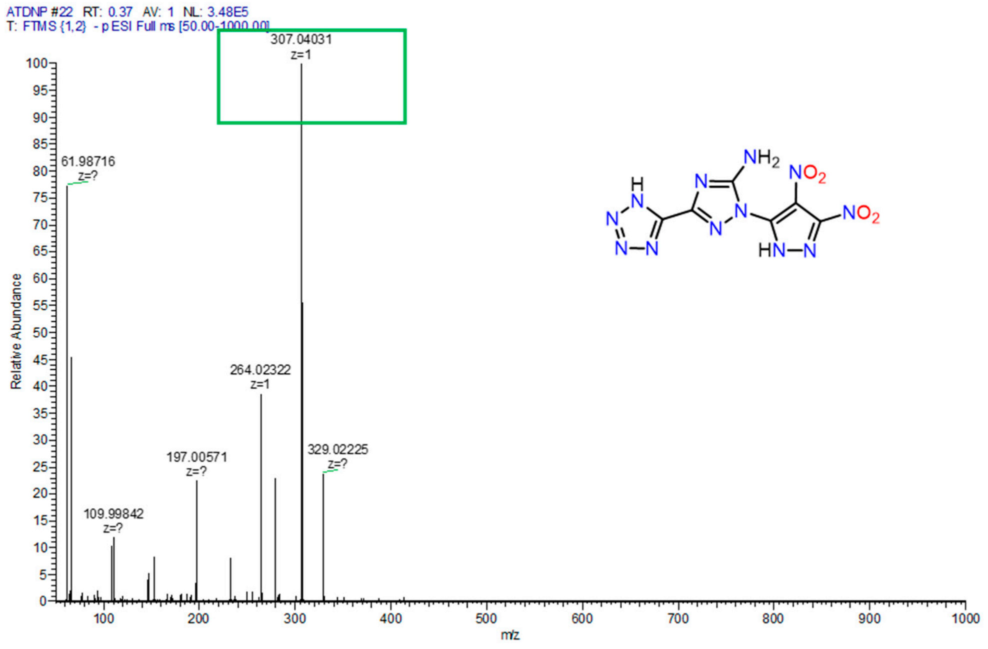

Figure S16. HRMS spectrum for **ATDNP**

5. IR spectra of all new compounds

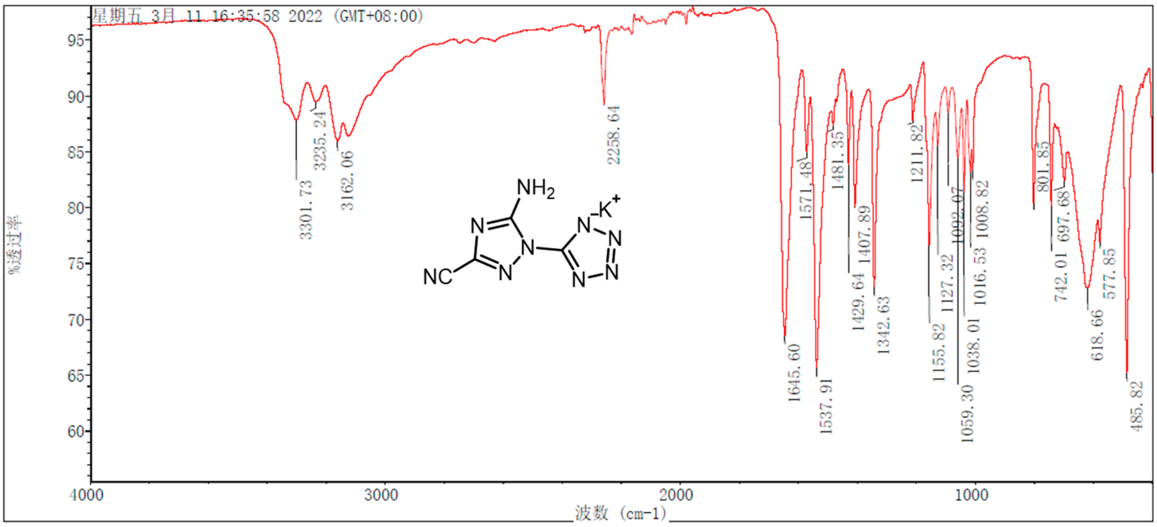

Figure S17. IR spectrum of compound **1**

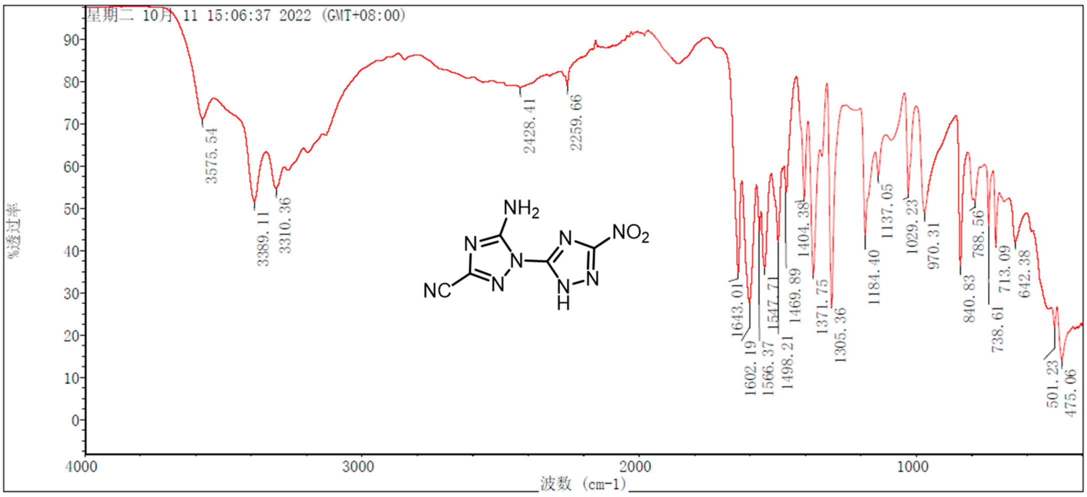

Figure S18. IR spectrum of compound **2**

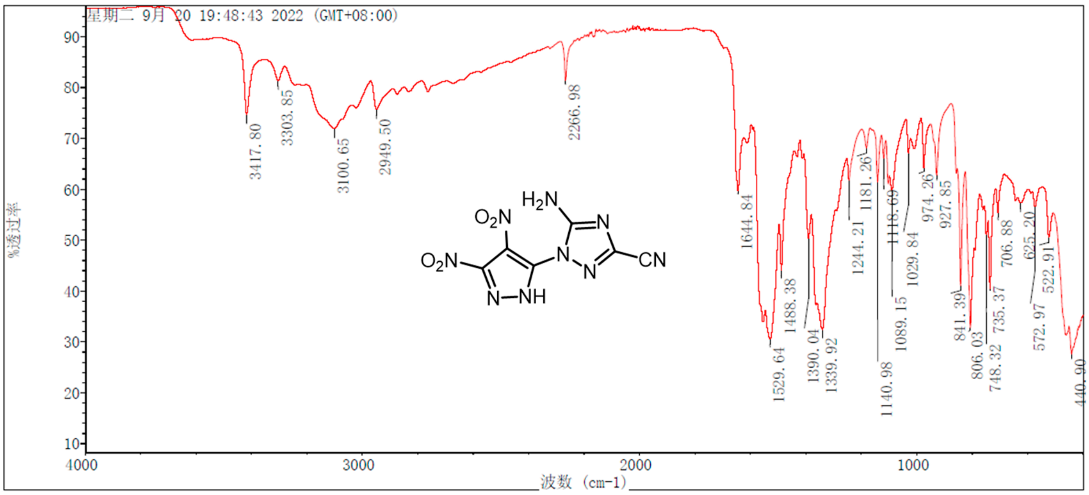

Figure S19. IR spectrum of compound **3**

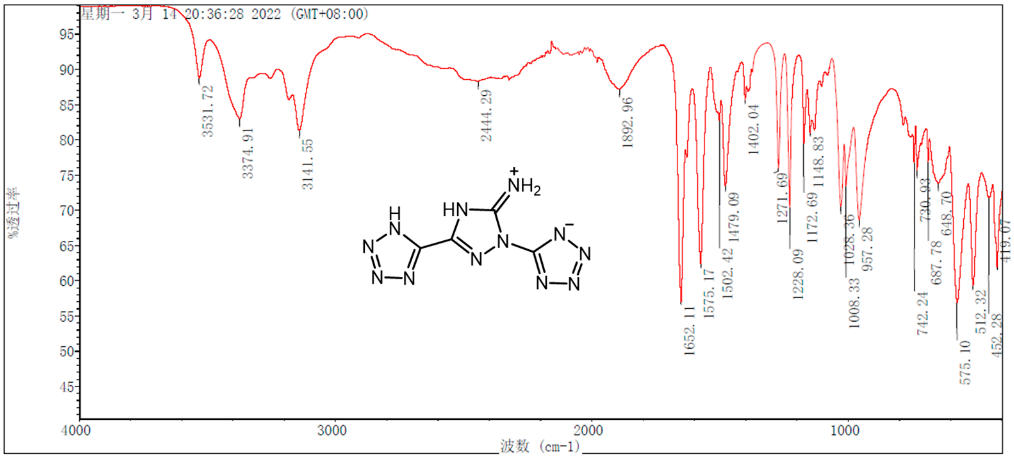

Figure S20. IR spectrum of compound **ATDT**

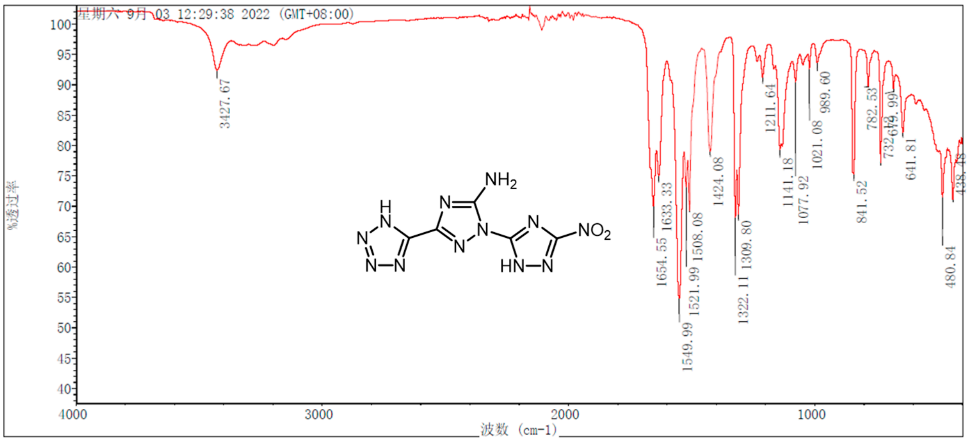

Figure S21. IR spectrum of compound **ATNT**

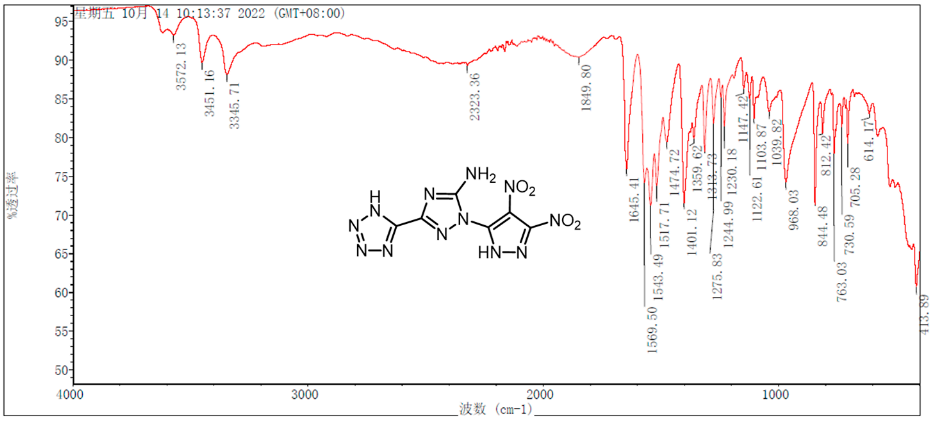

Figure S22. IR spectrum of compound **ATDNP**

6. DSC plots for ATDT, ATNT and ATDNP

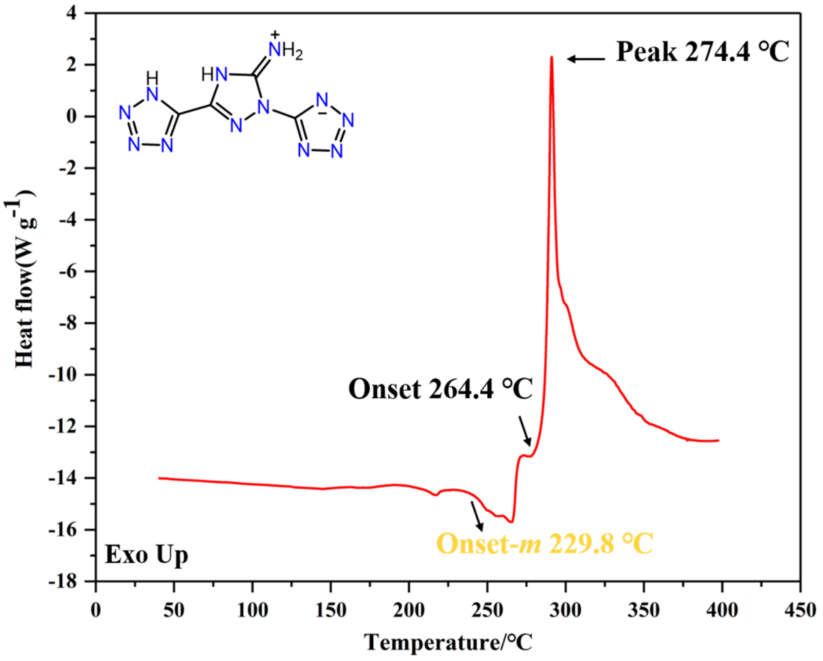

Figure S23. DSC curve of compound **ATDT** at 5 °C min^{-1}

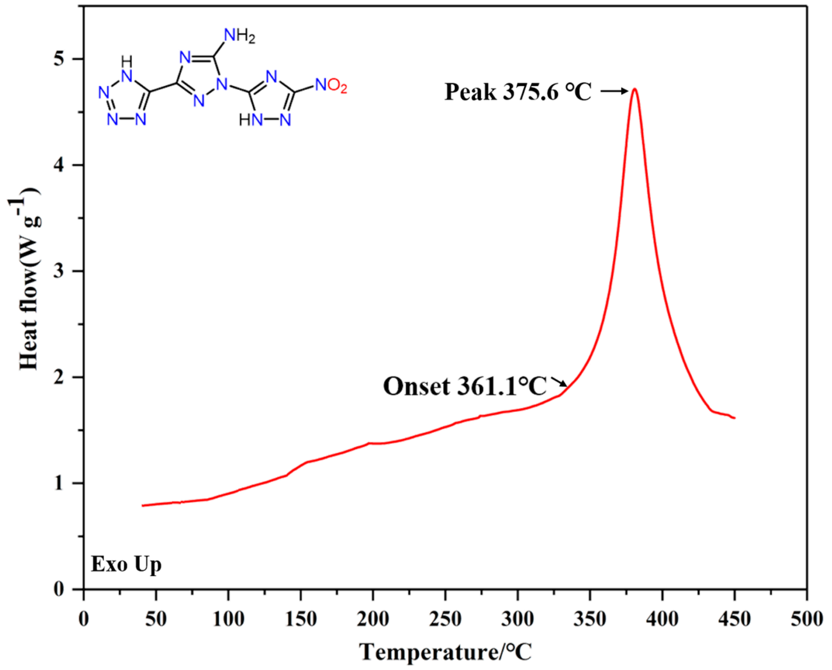

Figure S24. DSC curve of compound **ATNT** at 5 °C min^{-1}

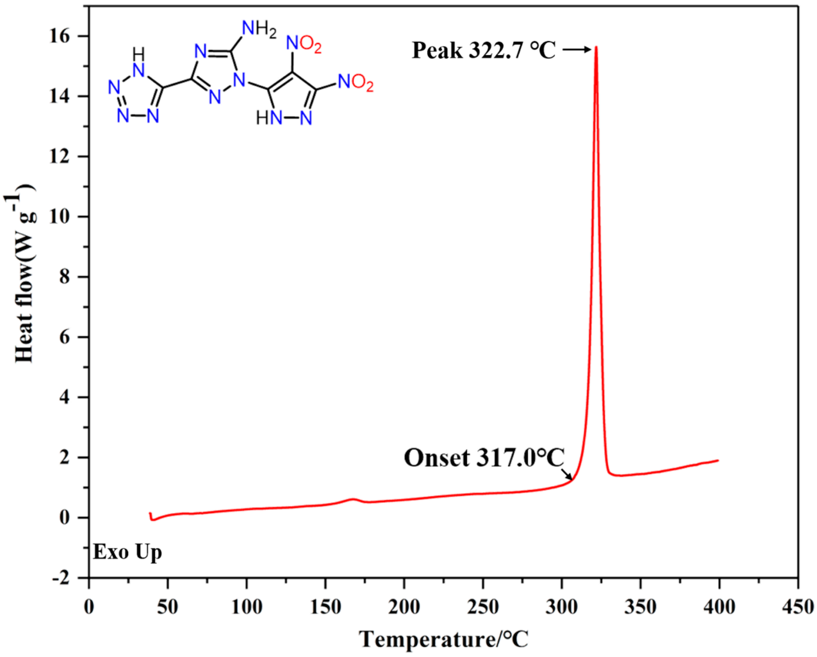

Figure S25. DSC curve of compound **ATDNP** at 5 °C min^{-1}

References

[1] (a) Strömberg, A.; Gropen, O.; U. Wahlgren, Gaussian basis sets for the fourth-row main group elements. In–Xe, *J. Comput. Chem.* **1983**, *4*, 181–186; (b) Glukhovtsev, M. N.; Pross, A.; McGrath, M. P.; Radom, L. Extension of Gaussian-2 (G2) theory to bromine-and iodine-containing molecules: Use of effective core potentials. *J. Chem. Phys.* **1995**, *103*, 1878–1885.

[2] M. S. Westwell, M. S. Searle, D. J. Wales, D. H. Williams, Empirical Correlations between Thermodynamic Properties and Intermolecular Forces. *J. Am. Chem. Soc.* **1995**, *117*, 5013–5015.
